# Development and Characterization of Peruvian Native Potato Starch/PVA-Based pH-Sensitive Films Incorporated with Purple Potato Anthocyanin Extract for Food Packaging

**DOI:** 10.3390/polym17131813

**Published:** 2025-06-29

**Authors:** Leandro Neodini Remedio, Carolina Parada-Quinayá

**Affiliations:** 1 Centro de Investigación en Bioingeniería, Universidad de Ingenieria y Tecnologia UTEC, Jr. Medrano Silva 165, Lima 15063, Peru; dparada@utec.edu.pe; 2 Bioengineering and Chemical Engineering Department, Universidad de Ingenieria y Tecnologia UTEC, Jr. Medrano Silva 165, Lima 15063, Peru

**Keywords:** potato starch, anthocyanin, intelligent film, pH indicator

## Abstract

Intelligent films (IFs) incorporating natural colorants and biodegradable materials offer innovative solutions for monitoring food freshness and spoilage. This study evaluated the impact of varying the PVA-APN ratio on films formulated with Peruvian Purple Potato starch (APN) and anthocyanin extract (AE). The research focused on the effects of PVA on physicochemical and mechanical characteristics, as well as the color changes observed when the films were used with seafood. The results indicated a decrease in chroma a* and an increase in chroma b* when the films were in contact with different buffer solutions (from acidic to alkaline). Solubility decreased with higher starch concentrations and the mechanical properties revealed a reduced tensile strength and elongation with increased APN concentration. The films effectively indicated freshness, with the best Δ*E* values for the 50:50 formulations (13.6 ± 1.6 and 12.04 ± 2.8 for fish and shrimp, respectively), making them promising candidates for intelligent seafood packaging.

## 1. Introduction

The widespread use of single-use plastic food packaging has significantly increased in recent years, driven by factors such as convenience, extended shelf life, and mass production demands. This growing reliance on synthetic films has exacerbated environmental concerns due to their non-degradable nature, contributing to the global plastic waste crisis [1]. A promising approach to addressing this issue is the development of biodegradable food packaging made from materials such as polysaccharides, proteins, or biopolyesters like polyvinyl alcohol (PVA) and polylactic acid, which are affordable and frequently sourced from renewable materials or industrial by-products. To reduce plastic waste, food packaging plays a crucial role by ensuring the safety and quality of products. This can be achieved through the use of smart packaging, which monitors critical parameters such as temperature, pH, humidity, and total volatile basic nitrogen (TVB-N) levels [2].

The use of starch from different sources for film production has been studied extensively for decades. In the literature, smart films have been developed using starch derived from potato [3,4,5,6], corn [7,8,9,10], cassava [11,12,13,14], rice [15], and other sources. However, despite their advantageous characteristics, such as excellent transparency [16,17], desirable organoleptic properties, and effective gas barrier capabilities [18,19], starch-based films have notable drawbacks, including low water resistance [20,21] and low mechanical properties [22,23]. To overcome these limitations and enhance the final properties of starch-based films, several strategies have been employed, such as blending with other polymers, optimizing the plasticizer content, and chemical cross-linking, all of which contribute to improving their mechanical strength, barrier performance, and overall suitability for food packaging applications [6,24].

Polyvinyl alcohol (PVA) is a polymer extensively used in food packaging due to its desirable properties, including biodegradability, non-toxicity, chemical resistance, low oxygen permeability, and excellent film-forming capacity [25], and the production of blends combining starch and polyvinyl alcohol (PVA) is frequently reported in the literature, aiming to harness the benefits of both polymers. Analyzing cassava starch/PVA films for smart film production, Wu et al. [12] reported on films exhibiting the best mechanical properties with a maximum tensile strength of 23.07 MPa and an elongation at break of 291.22%. This occurs mainly because the gelatinized starch granules can be incorporated into the PVA matrix, forming strong bonds with one another. An increase in PVA content promotes the formation of a more stable and homogeneous mixed system, with hydrogen bonding serving as the primary mode of interaction between starch and PVA [26].

To develop intelligent films that offer easily observable visual responses, anthocyanins, natural pigments that belong to the family of phenolic compounds, are frequently used due to their ability to change color in response to varying pH levels. These color-changing compounds can be sourced from a variety of natural products, including red cabbage [27,28,29], purple sweet potato [30,31,32], black carrot [33,34], blueberry [5], mulberry [35] and eggplant [36], and consistently exhibit color variations when applied to different foods, demonstrating their effectiveness in producing smart films. Previous studies have shown that anthocyanin extracts from the Peruvian purple potato “INIA 328–KULLI” exhibit significant color variation across different pH levels, ranging from dark red at pH 1 to green at pH 10 [7]. While many studies have explored films based on starch, PVA, and anthocyanins, this research stands out for using a single native Peruvian raw material—the purple potato—as the exclusive source for both the starch and the anthocyanin extract. This integrated use may enhance film compatibility and promote local sustainability through the valorization of a regional resource.

Therefore, this study aimed to evaluate the effect of varying concentrations of polyvinyl alcohol (PVA) on films produced from starch (APN) and anthocyanin extract (AE) obtained from the Peruvian Purple Potato “INIA 328–KULLI”. The influence of PVA on the films’ physicochemical and mechanical properties was analyzed, along with their color response after application to seafood.

## 2. Materials and Methods

### 2.1. Materials

Starch (APN) and anthocyanin extract (AE) obtained from the extraction of Peruvian purple potato “INIA 328–KULLI” according to previous work published by Neciosup-Puican et al. [7], polyvinyl alcohol (PVA) purchased from Sigma-Aldrich (Mw 89,000–98,000, >99% hydrolyzed, St. Louis, MO, USA) and sorbitol purchased from RandhuLab (Lima, Peru) were used to produce the films, and hydrochloric acid (37%, lote:C28W32, JT Baker, Avantor Performance Materials, Phillipsburg, NJ, USA) was used to adjust the pH of the filmogenic solution. For the stability tests, the following salts were used: Lithium chloride (purity ≥ 99%, M = 42.39 g/moL, CDH, Central Drug House, New Delhi, India); Magnesium chloride (purity ≥ 99%, M = 95.21 g/moL, CDH, Central Drug House, New Delhi, India); Sodium chloride (purity ≥ 99.5%, M = 58.44 g/moL, Oxford Lab Fine Chem LLP, Mumbai, India); and Potassium chloride (purity ≥ 99.5%, M = 74.55 g/moL, CDH, Central Drug House, New Delhi, India). For the color tests, standard pH buffers were used: pH 1.68 (Lot: 4GA1203, OHAUS Corporation, Parsippany, NJ, USA); pH 4.01 (Lot: 4GA1251, OHAUS Corporation, Parsippany, NJ, USA); pH 7.00 (Lot: 4GC0941, OHAUS Corporation, Parsippany, NJ, USA); pH 10.01 (Lot: 4GA0900, OHAUS Corporation, Parsippany, NJ, USA); and pH 12.45 (Lot: 8744, Hanna Instruments, Inc., Woonsocket, RI, USA).

### 2.2. Production of Intelligent Films (IFs)

PVA and APN films were developed using the tape-casting technique, using the following proportions of PVA and APN for each polymer: 100:0 (FPVA); 75:25 (FAPN25); 50:50 (FAPN50); 25:75 (FAPN75) and 0:100 (FAPN100). Two separate solutions were prepared, one containing APN and the other containing PVA. These solutions were then mixed, and anthocyanin extract (AE) was added at a concentration of 5%. For the APN solution, the starch, at a concentration of 7 g/100 g film-forming solution (FS), was initially dissolved in distilled water with sorbitol (10 g/100 g of polymer) and HCl (0.2 M) under magnetic stirring (300 rpm) for 10 min. The solution was heated for 20 min at 90 °C and 600 rpm to promote its gelatinization. In parallel, a PVA solution was produced, where PVA, at a concentration of 10 g/100 g FS, was dissolved in distilled water for 3 h at 500 rpm and 70 °C. The two solutions were mixed in their proper proportions and kept under magnetic stirring at 500 rpm for 60 min, followed by an ultrasonic bath (VEVOR JPS-10A, 60 W, Rancho Cucamonga, CA, USA) for 30 min at 60 °C. Then, 1 g of AE/100 g FS was added under constant stirring. The final solution was spread on an acrylic plate using the tape-casting technique (Film Coater MSK-AFA-III, Shenyang Kejing Auto-Instrument Co., Ltd., Liaoning, China), with a thickness of 2000 μm and spreading speed of 10 mm/s. It was then dried in an oven at 40 °C for 8 h. All films obtained were stored in a desiccator with NaBr (RH = 58%) for 7 days before characterization.

### 2.3. Characterization of IFs

#### 2.3.1. Visual Aspect and Color Parameters

The visual appearance of the films was evaluated in relation to the presence of particles, bubbles, the formation of a continuous matrix, the ease of removal from the acrylic plates and the absence of deformation, and the uniform distribution of the AE on the IF surface.

The color parameters were determined according to Wang et al. [37] using the PCE-XXM 30 colorimeter (PCE Instruments, Manchester, UK), which generated values in the CIELab color spectrum. Film samples (1 cm × 1 cm) were submerged in different buffer solutions (pH 1.68; 4.01; 7.00; 10.01; 12.45) for 30 min. Films were then removed from the solution, dried at room temperature and analyzed. The parameters chroma a*, chroma b*, and L* (luminosity) were observed. In addition, the parameters chroma c*, h* and Δ*E* were calculated according to Equations (1)–(5) [38]:

(1) $$chroma\;c^{*}=\sqrt{{{\left(a^{*}\right)}^{2}+\left(b^{*}\right)}^{2}}$$

(2) $$h={tan}^{-1}\left(\frac{b^{*}}{a^{*}}\right)$$

if a* and b* are positive.

(3) $$h={180+tan}^{-1}\left(\frac{b^{*}}{a^{*}}\right)$$

if a* and b* are negative, or if a* is negative and b* is positive.

(4) $$h_{ab}={360+tan}^{-1}\left(\frac{b^{*}}{a^{*}}\right)$$

if a* is positive and b* is negative.

(5) $$\Delta E=\sqrt{{\left(L_{0}-L^{*}\right)}^{2}+{\left(a_{0}-a^{*}\right)}^{2}+{\left(b_{0}-b^{*}\right)}^{2}}$$

where the values of *L*_0_, *a*_0_ and *b*_0_ are the values at the initial moment; and L*, a* and b* are these parameters at the moment to be determined. The L* parameter represents the luminosity of the films, while the chroma a* and chroma b* parameters represent the color shifts from green to red and from blue to yellow, respectively. The chroma c* values indicate the degree of color saturation, h* represents the dominant hue in the spectrum, and Δ*E* reflects the total color change of the film compared to their initial color before being exposed to buffer solutions at different pH levels.

#### 2.3.2. Moisture Content and Solubility

The IF moisture content was determined using a thermogravimetric balance (MB120-Ohaus) according to AOAC [39]. Samples (0.5 g) were added to the equipment and dried at 105 °C to determine the mass loss of IFs until it remained constant.

The solubility was determined according to the methodology proposed by Gontard et al. [40] after 24 h in distilled water. Initially, the film samples (20 mm × 20 mm) were immersed in 50 mL of distilled water with constant stirring (100 rpm) using a shaker (Shaker MA-420-Marconi, São Paulo, Brazil) for 24 h at 25 °C. Then, the samples were dried at 105 °C. Solubility was determined by Equation (6) [40]:

(6) $$S=\frac{\left|m_{i}-m_{f}\right|}{m_{i}}\times 100$$

where *S* is solubility in water (g/100 g of IFs); *m_i_* is the initial mass (g); and *m_f_* is the final mass after immersion in water and drying (g).

#### 2.3.3. Mechanical Properties

The mechanical properties of the IFs, tensile strength (TS), elongation (ε) and Young’s modulus (ME) were determined according to ASTM D882-10 (ASTM International, 2012) using a TA.XT Plus texturometer (TA Instruments, New Castle, DE, USA), with the test conditions set at 100 mm for the initial separation distance and test speed set at 1.0 mm/s.

#### 2.3.4. Contact Angle

The contact angle of the films was determined by depositing a droplet of deionized water (5 μL) onto each film sample (30 mm × 20 mm) and measured using the Attension Theta Lite tensiometer (KSV Instruments, Biolin Scientific AB, Västra Frölunda, Sweden). The behavior of the water droplet deposited on the film was assessed at the initial moment and after 30 s of deposition, following the methodology proposed by Shahbazi et al. [41].

#### 2.3.5. Scanning Electron Microscopy (SEM)

The surface and internal structure of the IFs were evaluated using a TM-3000 electron microscope (Hitachi, Tokyo, Japan). For external surface analysis, the samples (20 mm × 20 mm) were fixed with double-sided adhesive tape and analyzed using a 5 kV electron beam. To analyze the internal structure, they were immersed in liquid nitrogen and then fractured and analyzed using a 15 kV electron beam. Before analysis, the IFs were kept in a desiccator containing silica gel (10 days, 25 ± 2 °C).

#### 2.3.6. Fourier Transform Infrared Spectroscopy (FTIR)

Fourier transform infrared spectroscopy (FT-IR) was used to determine the structural interactions between the components present in the matrix. A Pekin Elmer spectrophotometer with an attenuated total reflectance (ATR) accessory (Spectrum One, Rocklin, CA, USA) was used. Prior to analysis, the film samples (20 mm × 20 mm) were stored in desiccators with silica for a period of 7 days at room temperature (25° ± 2 °C). A total of 32 scans (n = 32) were used as a parameter in a spectral range ranging from 650 to 4000 cm^−1^.

#### 2.3.7. X-Ray Diffraction (XRD)

The crystallinity of the IFs was determined using a MiniFlex 600 diffractometer (Rigaku, Tokyo, Japan) operating at 40 kV and 15 mA with a Cu-Kα point source. The scanning speed was 1°/min with a step of 0.02° for each scan, which was performed between 5° and 50° (2θ). Samples (20 mm × 20 mm) of the IFs were previously stored in a desiccator containing silica for a period of 7 days at room temperature (25 ± 2 °C).

#### 2.3.8. Color Stability

To analyze the color stability, the films were preserved in amber desiccators and kept in the absence of light, with a constant relative humidity maintained by saturated salt solutions, following the methodology proposed by Guo et al. [42]. The samples were conditioned for a period of three months at room temperature (25 ± 2 °C). The following salts were used: lithium chloride (LiCl = 11.15% RH), magnesium chloride (MgCl_2_ = 32.73% RH), sodium bromide (NaBr = 57.70% RH), sodium chloride (NaCl = 75.32% RH), and potassium chloride (KCl = 84.32% RH). Color parameters *L*, a*, and b* were calculated every 7 days for a total analysis period of 60 days, following the methodology described in Section 2.3.1, for pH levels of 4.01, 7.00, and 10.01.

#### 2.3.9. Application of pH-Responsive Films in Monitoring Food Freshness

Shrimp and trout fillet, purchased from a local market (Lima, Peru), were used to detect the color change response of the films following the methodology proposed by Mohammadalinejhad [43]. Film samples (1 cm × 1 cm) were placed on the inner surface of a petri dish without direct contact with the food. The petri dishes were sealed with parafilm and stored at 25 ± 2 °C for 3 days. The color changes occurring in the films were analyzed every 24 h using the parameters listed in Section 2.3.1.

### 2.4. Statistical Analysis

All formulations were produced in triplicate and, for each analysis performed, a triplicate determination of each replicate was made. For the mechanical properties, a total of 10 samples were used for each replicate. All results were statistically verified using InfoStat software (Version 5.13.1) by one-way analysis of variance (ANOVA), using the Tukey test with 95% confidence.

## 3. Results and Discussion

### 3.1. Production of Intelligent Films

Starch-based films produced by the tape-casting technique, containing different concentrations of PVA, showed flexibility, homogeneity and an absence of insoluble particles, exhibiting the formation of a continuous matrix and a dark pink color evenly distributed due to the presence of anthocyanin extract (AE). The distribution of the AE over the visually uniform surface and the films exhibited controlled thickness. Except for the formulation containing only APN (FAPN100), all formulations had a thickness ranging from 0.102 to 0.125 mm.

### 3.2. Characterization of IFs

#### 3.2.1. Visual Aspect and Color Parameters

Table 1 shows the color parameters of the films under different pH conditions. Films with higher concentrations of PVA exhibit increased L values, indicating greater lightness. This can be attributed to the smoother surface and higher transparency of PVA, which tends to reflect more light compared to starch. Starch-based films, in contrast, are typically opaquer and have a rougher surface, which scatters light, resulting in lower L values.

The glossy nature of PVA contributes to its ability to produce films with a brighter appearance. In general, for all formulations, regardless of the proportion used, a decrease in a* and an increase in b* were observed. This indicates that, as the pH increased, the films exhibited a tendency to change their colors, transitioning from shades closer to red at pH 1.68, to light pink at pH 7.00, green when in contact with buffer at pH 10.01, and finally dark yellow at pH 12.45.

The chroma c* parameter measures the color saturation or intensity of the films. Higher c* values are associated with more vivid and saturated colors, while lower values are indicative of less intense colors. It was observed that the c* values varied with changes in pH, reflecting the shifts in color intensity across different pH levels.

Regarding the total color change (Δ*E*), it was observed that all films showed an increase in Δ*E* values as the pH of the solution increased. This indicates that the films underwent a more pronounced color shift with higher pH levels. The increase in Δ*E* reflects a greater overall change in color, suggesting that the film’s color response is sensitive to pH variations. As the pH increased, the films demonstrated a more significant deviation from their initial color, highlighting their effectiveness as pH-sensitive indicators.

Similar results were observed by Choi et al. [6] in potato starch films with anthocyanin extract, where a* values decreased from 32.25 ± 0.54 to −2.67 ± 0.30 as the pH increased from 2 to 10. In intelligent myofibrillar protein films with anthocyanins, Zheng et al. [44] reported a* values decreasing from 19.67 to 0.00, and b* from 5.00 and −1.00 as the pH increased from 3 to 12 for films with higher concentrations of the extract. Additionally, the Δ*E* values ranged from 5.23 to 17.08 with an increase in pH. In contrast, Chavez-Marquez et al. [4] observed a color change from red to brown in starch-based smart films with an increase in pH from 2 to 12. This change corresponded to a decrease in the a* and b* parameters from 45.20 ± 0.83 to 1.69 ± 0.43 and from 17.69 ± 0.88 to −3.92 ± 0.11, respectively, with Δ*E* values reaching up to 62.36. According to the authors, Δ*E* values above 10 represent a color variation visible to the human eye.

**Figure 1:**
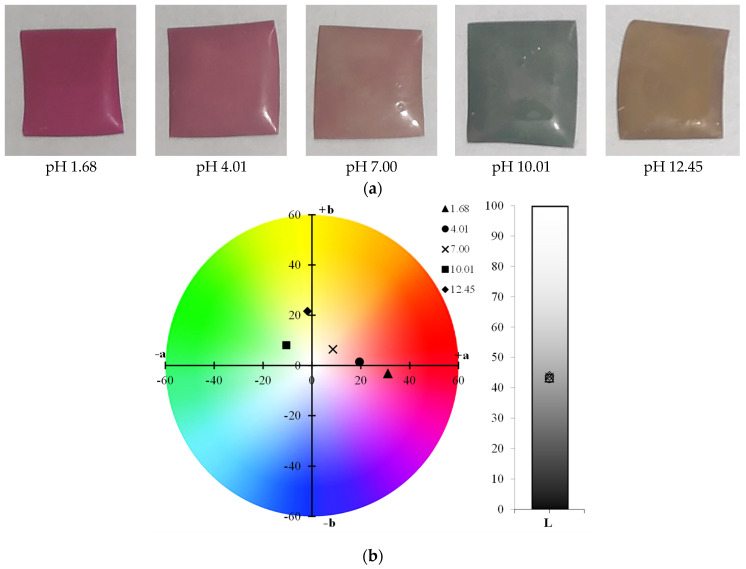
(**a**) Visual appearance of films after contact with buffer solutions, and (**b**) colorimetric variation in ab Parameters.

In Figure 1, the visual appearance of the films after contact with each of the different buffer solutions can be observed, along with the color variation measured using a colorimeter for the chroma a*, chroma b*, and luminosity L* parameters. A clear color variation in the films is evident, ranging from dark pink in films exposed to pH 1.68, transitioning to lighter tones as the pH increases, and reaching a green hue at pH 10.01 and a yellow hue at pH 12.45 (Figure 1a). Additionally, the color change in the films is clearly noticeable when plotted on the ab graph (Figure 1b), showing the transition from reddish-pink hues to yellow tones based on the colorimeter measurements. As previously described in the literature, Δ*E* values greater than 10 indicate color variations that are perceptible to the naked eye [45]. Thus, it can be stated that the films exhibited excellent color variation when in contact with different buffer solutions.

#### 3.2.2. Moisture Content and Solubility

The effect of the PVA on the moisture content and solubility of the films are presented in Table 2. No significant differences in the moisture content were observed between the films, regardless of the PVA concentration. Starch and PVA-based films can vary in moisture content depending on several factors, such as the formulation, processing conditions, and environmental factors, with the moisture content typically reported in the literature ranging from 5% to 15% [1]. According to Liu et al. [46], the cross-linking of starch and PVA reduced the moisture sensitivity of the film by increasing polymer interactions within the blend, thereby decreasing the matrix’s affinity for water and reducing its hydrophilic nature. Qin et al. [47], analyzing starch and PVA-based films with anthocyanins from *Lycium ruthenicum*, observed a moisture content ranging from 8.72 ± 0.09% to 11.44 ± 0.48% for films with and without anthocyanins, respectively. According to the authors, the strong interactions between water molecules and the hydrophilic hydroxyl groups formed in the starch–PVA mixture caused the reduction in moisture content.

The use of different concentrations of PVA caused significant differences in the solubility of the films. Increasing the proportion of APN decreased their solubility from 98.4 ± 0.3% to 35.1 ± 3.5%, representing a reduction of approximately 65%. This decrease may be due to the high solubility of PVA in the samples. Patil et al. [48] found that incorporating PVA into starch-based films increased the water solubility, ranging from 29.5% for films with 10% PVA to 89.9% for films made with pure PVA. Films with a lower PVA content exhibited reduced solubility due to their increased water resistance. The presence of hydroxyl groups (–OH) in PVA contributes to higher water absorption, swelling, and solubility, leading to greater solubility compared to films with lower PVA proportions [1].

#### 3.2.3. Mechanical Properties

**Figure 2:**
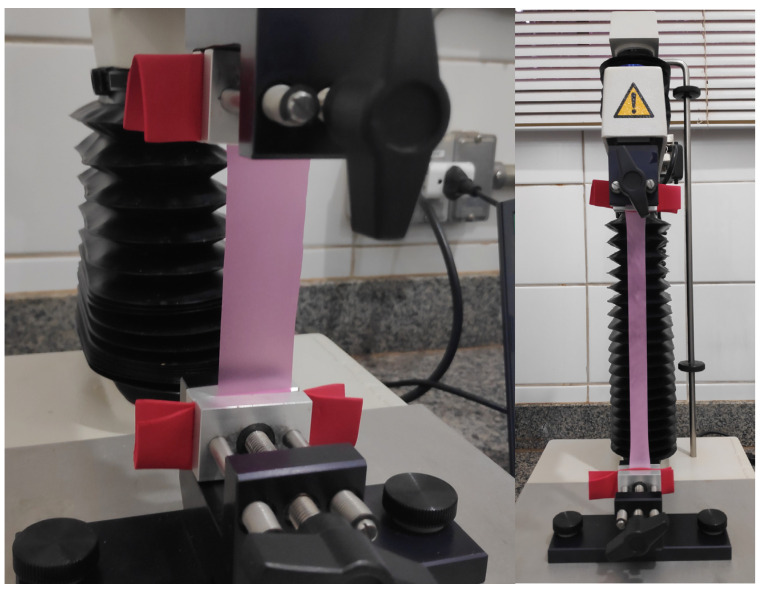
Texture analyzer testing of FAPN25 film for mechanical property assessment.

The tensile strength (TS), elongation (ε), and Young’s modulus of the films are presented in Table 3. The addition of APN caused a significant reduction in tensile strength (TS), which decreased from 40.6 ± 6.8 MPa to approximately 15 MPa. However, except for the films made with APN alone (FAPN), there were no significant differences in the tensile strength (TS) values among the PVA:APN blends. To visually illustrate the mechanical test setup, Figure 2 shows the FAPN50 film during the tensile test performed using the texture analyzer.

The elongation results indicate a substantial decrease when APN was present in the films, showing a decrease from 277.6 ± 42.5% to 2.6 ± 0.9% with an increasing APN concentration. Patil et al. [48] analyzed the effect of adding different concentrations of PVA to starch-based films and found that films containing only PVA exhibited a higher tensile strength and elongation compared to those with starch alone, ranging from 17.9 to 2.3 MPa and 27.4 to 219.8%, respectively. For 50:50 PVA–starch blends, the tensile strength was approximately 10 MPa and the elongation around 70%. Despite covalent bonding, the polymer chains in the PVA–starch composite films retain significant chain flexibility. This enhancement in mechanical properties is attributed to the high mechanical strength of PVA, owing to its flexible C-C backbone structure and the abundance of –OH groups. Wu et al. [12], analyzing starch and PVA-based films incorporated with 7% anthocyanins from *Aronia melanocarpa*, found tensile strength and elongation values of 23.07 MPa and 291.22%, respectively. According to the authors, these values indicate strong mechanical properties, primarily due to the incorporation of gelatinized starch granules into the PVA matrix, which forms tight bonds between them.

The increase in APN results in a drastic rise in their Young’s modulus values. Young’s modulus measures a material’s resistance to elastic (recoverable) deformation under load, with higher values indicating stiffer materials and lower values indicating more flexible materials. These results support the elongation findings, suggesting that higher starch concentrations enhance the cohesiveness of the matrices, leading to increased rigidity and reduced elasticity.

#### 3.2.4. Contact Angle

Table 3 presents the values obtained for the contact angle of the films at the initial moment of droplet deposition (t = 0 s) and after 30 s. It can be observed that there was no variation in the contact angle after the initial 30 s of deposition for any of the formulations analyzed. However, a different effect was observed when analyzing the impact of different starch proportions in the formulations. FPVA exhibited the lowest contact angle values. When the lowest proportion of starch was added (FAPN25), a significant increase in these values was observed, likely due to the initial distribution of starch in the polymeric matrix. As the starch proportion increased, the contact angle values began to decrease. This may be attributed to a greater distribution of both starch and PVA on the film surface, enhancing its hydrophilic character, which facilitates water absorption and, consequently, reduces the contact angle. According to Hiremani et al. [49], in PVA films incorporated with oxidized corn starch, contact angles of 54.5° were observed for films with only PVA, while mixtures with starch exhibited a range from 61.4° to 91.4°. The authors indicated that PVA films exhibited highly hydrophilic behavior due to the stronger interaction between water and PVA. Moreover, the increase in the contact angle with the addition of starch could be attributed to the interaction between oxidized starch and PVA, which reduces the number of hydrophilic groups in the PVA film. According to Nizam et al. [50], the blending of starch with PVA reduced the hydrophilicity of the matrix, likely due to the formation of polymeric interactions and a reduced affinity for water. The hydrogen bonding between the hydroxyl groups of PVA and amylose or amylopectin causes the hydrophobic regions of the PVA chain to reorganize within the matrix, forming hydrophobic regions that lower the water affinity of the blended film.

#### 3.2.5. Scanning Electron Microscopy (SEM)

Table 4 presents images obtained from the scanning electron microscope (SEM) analyses, including both surface and internal structures. The FPVA and FAPN100 formulations exhibited smooth surfaces with no undissolved particles. In contrast, all other blends displayed similar characteristics, including low uniformity and a rough surface. This is likely due to the presence of PVA, which can form polymer networks and create complex interactions with other components.

These interactions may alter the internal structure of the matrices, leading to non-uniform polymer distribution and, consequently, a less uniform surface. This phenomenon is consistent with the observations made by Gómez-Bachar et al. [51] in starch/PVA blend films. According to the authors, the use of PVA induces a change in the morphology of the samples, making them rougher, which suggests a tough fracture due to the characteristics of PVA.

#### 3.2.6. Fourier Transform Infrared Spectroscopy (FTIR)

**Figure 3:**
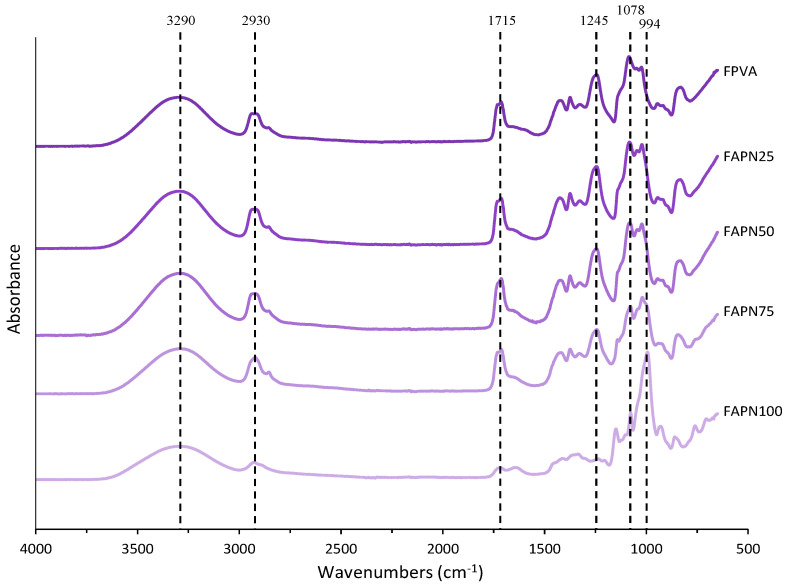
FTIR absorbance spectra for films with different PVA:APN ratios.

The FTIR absorbance spectra for films with different PVA:APN ratios are shown in Figure 3. Characteristic bands and peaks corresponding to each polymer can be identified. In all formulations, a band in the region around 3200–3500 cm^−1^ is associated with the stretching vibrations of the –OH bond [52]. The peaks at 2930 cm^−1^ correspond to C–H stretching vibrations [53]. The peaks at 1715 cm^−1^ were associated with the C=O stretching of non-hydrolyzed vinyl acetate groups, the peak at 1425 cm^−1^ corresponded to C–H asymmetric stretching and symmetrical bending, and the peak at 1078 cm^−1^ was related to C–O–C and C–O–H asymmetric stretching [54]. 

Characteristic peaks were detected around 1375 cm^−1^, corresponding to the stretching vibration modes of C–O bonds [55]. The stretching vibration of C–O in C–O–H groups is represented by the bands at 1240 and 1082 cm^−1^ [56]. Additionally, a peak at 994 cm^−1^ was observed, associated with the C–O vibration of C–O–H groups, which indicates a strong bonding interaction between the PVA and starch [57]. The stretching peak of aromatic C–C bonds in anthocyanin has been confirmed to appear in the 1600–1700 cm^−1^ region [53]. With the increase in the proportion of starch, an increase in the characteristic wavelength 994cm^−1^ of starch is observed, a peak also observed by Wu et al. [12] in starch and PVA films containing *Aronia melanocarpa* anthocyanins.

#### 3.2.7. X-Ray Diffraction (XRD)

**Figure 4:**
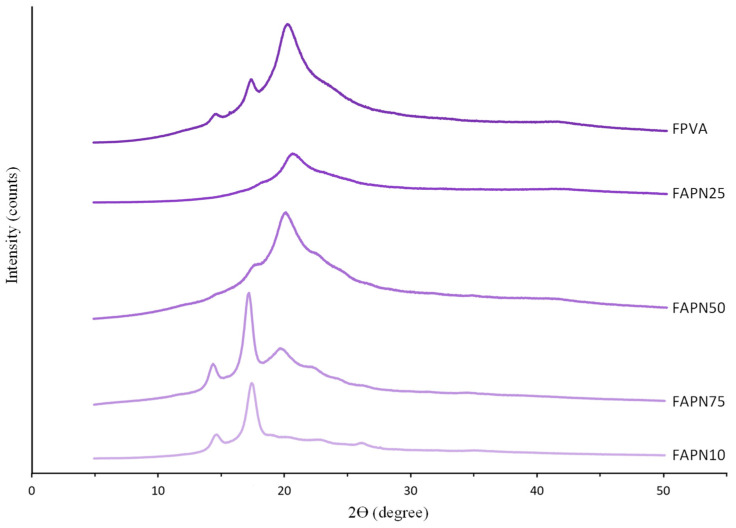
XRD patterns for films with different PVA:APN ratios.

The XRD patterns of the films are shown in Figure 4. The XRD patterns revealed that the starch/PVA-based films exhibited a combination of amorphous behavior and a semicrystalline structure, with characteristic peaks at 2θ = 15.0, 17.1, and 20.2°. An increase in the APN concentration led to a reduction in the intensity of the peak at 2θ = 20.2°, commonly associated with the crystalline domains of PVA. Concurrently, the appearance of broader peaks at 2θ = 15.0° and 17.1° suggests an enhanced contribution of amorphous starch regions, indicating a reduction in the overall crystallinity of the film matrix. Similar results were observed by Zheng et al. [58] in starch films, where peaks were detected in the region of 2θ = 15.0°, 17.0°, and 22.3°. In starch and PVA-based films with anthocyanins, W. Wu et al. [45] observed characteristic peaks at 2θ = 19.9° and two weak diffraction peaks at 2θ = 12.1° and 41.3°, which were attributed to PVA. The observed reduction in crystallinity with increasing APN content, indicated by the decrease in the PVA crystalline peak at 2θ = 20.2° and the increased amorphous starch regions, corresponds to the mechanical behavior described in Section 3.2.3. Specifically, films with higher APN concentrations exhibit increased Young’s modulus values and decreased elongation, reflecting a stiffer and less flexible matrix. Thus, changes in the crystalline structure detected by XRD are directly related to the textural properties of the films.

#### 3.2.8. Color Stability

The results for each color parameter as a function of storage time are presented below for each formulation, under exposure to pH values of 4.01 (Table 5), 7.00 (Table 6), and 10.01 (Table 7). At pH 4.01, the a* and b* parameters remained around 20 and 3, respectively, which are characteristic of a reddish coloration throughout the entire analysis period. For films subjected to buffer solutions at pH 7.00, the a* parameter values approached below 10, indicating a slight shift toward a yellowish tone, albeit with minimal changes in the b* parameter. Finally, when exposed to pH 10.01, the films exhibited a more drastic decrease in the a* parameter, dropping to values close to −10, while the b* parameter experienced a slight increase, reaching values near 10, representing a shift toward greener tones in the films.

However, no significant differences were observed concerning the different relative humidity levels over the analysis period. Regardless of storage time, all color analyses consistently showed statistically identical values for the L*, a*, and b* parameters. Similarly, the influence of the relative humidity on color parameters proved minimal, with no notable effects on any of the samples analyzed. This suggests that variations in storage conditions do not significantly affect the color stability of the films, reflecting the consistency of the formulations across different storage environments.

Wu et al. [45] evaluated the color stability of starch/PVA-based films incorporated with anthocyanins over a 30-day period at 25 °C and 57% relative humidity. The authors found that, after this period, all formulations exhibited minimal changes in both their appearance and color parameters, with the Δ*E* values remaining below 2 throughout the study. In starch and PVA-based films incorporated with roselle anthocyanins for monitoring fish freshness, Zhai et al. [59] demonstrated good stability over a 15-day storage period. When stored at 4 °C, the films exhibited a color variation of approximately 1% between Day 0 and Day 14. At 25 °C, the color change observed from the first to the last day of analysis was less than 5%. These findings underscore the films’ stability, regardless of the storage temperature.

#### 3.2.9. Application of pH-Responsive Films in Monitoring Food Freshness

Freshness-indicating labels can monitor the extent of spoilage in aquatic products such as shrimps and fish, which are rich in proteins and prone to spoilage, through changes in the color of the labels [60]. Because spoiled shrimp release volatile nitrides, creating an alkaline environment inside the package, IFs placed on the top of a package can detect this alkaline condition and exhibit color changes [61].

Table 8 and Table 9 show the changes in the color parameters (*L*, a*, b*, c*, h*, and Δ*E*) of the different films analyzed when used to test the freshness of fish and shrimp samples after 3 days of storage at 25 °C. Upon initiating the test, it was observed that the luminosity (L*) of all films changed during the first 24 h but remained constant as the storage period progressed, independent of the film type. However, it can be observed that for both FAPN50 and FAPN75 formulations, in both food samples analyzed, there is no significant variation in the a* and b* parameters during the first 24 h. However, after 48 h, a significant difference in these parameters is noticeable, with a decrease in a* from around 10 to near 2, and an increase in b* from −2 to values above zero, indicating a change in the final color of the film during the analyzed period. This color change is further reflected in the Δ*E*, which increases to approximately 10 for fish samples and around 8 for shrimp. Higher Δ*E* values indicate a more pronounced color change in the film, which may represent a visually significant and easily detectable alteration. After 72 h, the maximum Δ*E* values are observed for all films and both foods.

For the c* parameter, a decrease is observed as the food spoilage time increases. This reduction can be attributed to the sensitivity of the film to the volatile compounds released during spoilage, such as ammonia and other alkaline substances. These compounds cause a shift in the chroma of the film, reducing its color intensity and saturation. As a result, the decline in c* values reflects the film’s ability to detect changes in the environment, indicating the progressive deterioration of the food’s freshness. Similar results can be found in the literature for both fish and shrimp, confirming the effectiveness of using anthocyanins in the creation of IFs for monitoring food freshness, as observed by Li et al. [62], Huang et al. [2], Zheng et al. [44], and Mohammadalinejhad et al. [43].

**Figure 5:**
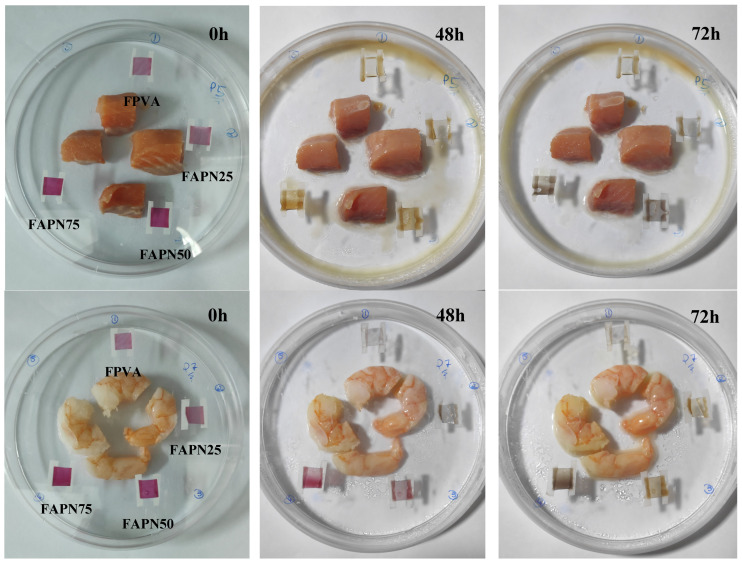
Color changes in the PVA:APN indicator film attached to packages with shrimp and fish, immediately after packaging and after 48 h and 72 h of storage at 25 °C.

In Figure 5, the color change in the films when exposed to the two food samples can be easily observed. On day 0, the films exhibit a dark pink color, characteristic of anthocyanins. For the fish sample, after 48 h, a visible color shift occurs, with the film turning to a dark yellow hue, which intensifies to shades close to blue after 72 h of decomposition. In the samples exposed to shrimp, the color change becomes more visually noticeable after 72 h, where the same dark yellow hue can be observed. These findings provide strong evidence that the films can effectively serve as intelligent indicators for detecting color changes in food, demonstrating their potential use as a reliable tool for monitoring the freshness of various food products through visible color shifts, which can be easily detected by consumers.

Poudel, Dutta, and Karak [63] produced starch-based films incorporated with anthocyanins from red hibiscus (*Hibiscus rosa sinensis*) petals and observed color variations in their formulations when exposed to raw fish samples at room temperature for a period of 72 h. According to the authors, the films initially exhibited a pink appearance on Day 0. However, after 24 h, they developed a dark purple color, followed by a greenish hue after 3 days. This color change clearly indicated a shift in the pH of the films, suggesting the release of volatile basic compounds resulting from the degradation of the raw fish.

Similar results were observed by Anugrah et al. [64] in alginate-based films incorporated with anthocyanins from red cabbage and zinc oxide nanoparticles when testing the freshness of prawns. The films exhibited a color change, shifting from dark pink to green after 24 h of exposure to the samples. Furthermore, color variations in the films were analyzed when exposed to the same samples frozen at −11 °C, with similar color changes observed after 5 days of storage. These findings highlight the significant potential of such films for industrial-scale applications in intelligent food packaging.

Wu et al. [45] also reported color variations in their starch/PVA-based films incorporated with anthocyanins, which changed from purple to gray and finally to dark green after 48 h of storage with shrimp samples at 25 °C and 25% relative humidity. Additionally, the authors correlated the observed color changes with the levels of Total Volatile Basic Nitrogen (TVB-N) in the environment. An increase in the TVB-N content (mg/100 g) was observed, ranging from 4.12 (0 h, fresh) to 22.68 (24 h) and 35.86 (36 h, deteriorated), which explains the color variation in the films and confirms their potential use as intelligent packaging.

## 4. Conclusions

In this study, an intelligent film made from Peruvian native potato starch (APN) and PVA, incorporated with anthocyanin extract, was successfully developed and characterized for food freshness monitoring. The results showed that chroma a* decreased while chroma b* increased with rising pH levels, indicating changes in color intensity and hue. The solubility decreased with higher APN concentrations, ranging from 98.4% to 35.1%. The mechanical properties revealed a decrease in the tensile strength and elongation with an increased APN content, which increased the cohesion of the matrices, leading to increased rigidity and reduced elasticity. The best mechanical performance was observed in FAPN50 and FAPN75, making them the most suitable for practical applications. SEM analysis showed rough surfaces for PVA:APN mixtures and smoother surfaces for single-component films, while FTIR and X-ray diffraction confirmed characteristic peaks, with an amorphous behavior and a semicrystalline state. Good stability was observed in the films over a 90-day period, with no significant differences in color parameters after this time, regardless of the formulation or the relative humidity of the environment. The films effectively demonstrated color changes when applied to seafood, with the greatest Δ*E* values observed in FAPN50 (13.60 ± 1.65 for fish and 12.04 ± 2.77 for shrimp), reinforcing its potential use as intelligent packaging for freshness detection. Consequently, the PVA:APN indicator film is an excellent candidate for innovative future packaging solutions aimed at monitoring seafood freshness.

## Figures and Tables

**Table 1:** Changes in color parameters of films under different pH conditions.

Formulation	pH	L*	a*	b*	c*	h*	Δ*E*
FPVA	1.68	57.8 ± 1.1 ^a^	39.1 ± 1.7 ^a^	−9.3 ± 0.2 ^a^	40.2 ± 1.6 ^a^	359.77 ± 0.01 ^a^	6.9 ± 1.0 ^a^
	4.01	64.7 ± 0.5 ^b^	20.2 ± 0.5 ^b^	−2.3 ± 0.2 ^b^	20.3 ± 0.5 ^b^	359.89 ± 0.01 ^a^	25.4 ± 0.6 ^b^
	7.00	69.5 ± 0.9 ^c^	10.6 ± 0.9 ^c^	5.2 ± 0.3 ^c^	11.8 ± 0.8 ^d^	0.45 ± 0.05 ^c^	38.2 ± 1.2 ^c^
	10.01	63.7 ± 1.2 ^b^	−11.9 ± 0.2 ^e^	8.1 ± 0.5 ^d^	14.4 ± 0.5 ^c^	179.40 ± 0.02 ^b^	55.4 ± 0.2 ^d^
	12.45	70.6 ± 0.5 ^c^	−0.7 ± 0.1 ^d^	22.2 ± 0.1 ^e^	22.2 ± 0.1 ^b^	178.46 ± 0.01 ^b^	55.4 ± 0.3 ^d^
FAPN25	1.68	68.9 ± 0.3 ^a^	9.5 ± 0.7 ^a^	1.4 ± 1.1 ^a^	9.6 ± 0.6 ^b^	0.15 ± 0.12 ^a^	28.9 ± 0.9 ^a^
	4.01	72.7 ± 1.2 ^b^	4.8 ± 0.3 ^b^	1.3 ± 0.4 ^a^	5.0 ± 0.2 ^d^	0.26 ± 0.09 ^a^	33.1 ± 0.9 ^b^
	7.00	70.7 ± 1.9 ^a,b^	5.1 ± 0.9 ^b^	5.0 ± 0.4 ^b^	7.1 ± 0.6 ^c^	0.78 ± 0.11 ^b^	33.3 ± 1.4 ^b^
	10.01	71.3 ± 0.5 ^a,b^	2.9 ± 0.9 ^c^	2.6 ± 0.5 ^a^	3.9 ± 0.9 ^d^	0.74 ± 0.10 ^b^	32.6 ± 0.7 ^b^
	12.45	76.8 ± 0.7 ^c^	−3.7 ± 0.1 ^d^	11.9 ± 0.3 ^c^	12.5 ± 0.3 ^a^	178.73 ± 0.01 ^c^	41.9 ± 0.1 ^c^
FAPN50	1.68	55.3 ± 3.7 ^a^	25.1 ± 1.3 ^a^	−4.5 ± 0.7 ^a^	25.5 ± 1.3 ^a^	359.82 ± 0.02 ^a^	17.6 ± 2.4 ^a^
	4.01	57.6 ± 1.4 ^a^	20.9 ± 0.6 ^b^	−2.2 ± 0.3 ^b^	21.0 ± 0.5 ^b^	359.89 ± 0.02 ^b^	25.4 ± 1.2 ^b^
	7.00	69.7 ± 0.2 ^b^	6.3 ± 0.1 ^c^	5.6 ± 0.1 ^c^	8.5 ± 0.1 ^d^	0.73 ± 0.01 ^e^	43.6 ± 0.1 ^c^
	10.01	59.4 ± 2.2 ^a^	−11.5 ± 0.2 ^e^	7.7 ± 0.4 ^c^	13.9 ± 0.4 ^c^	179.41 ± 0.01 ^c^	55.7 ± 0.8 ^d^
	12.45	58.5 ± 0.9 ^a^	−0.8 ± 0.2 ^d^	23.9 ± 1.6 ^d^	23.9 ± 1.6 ^a^	178.46 ± 0.01 ^d^	56.4 ± 0.9 ^d^
FAPN75	1.68	46.4 ± 1.0 ^b^	32.1 ± 0.6 ^a^	−0.5 ± 0.4 ^a^	32.1 ± 0.6 ^a^	359.04 ± 0.77 ^a^	9.6 ± 1.2 ^a^
	4.01	49.6 ± 0.6 ^b^	19.9 ± 1.0 ^b^	3.6 ± 0.2 ^b^	20.3 ± 1.0 ^b^	10.33 ± 0.09 ^e^	20.5 ± 1.1 ^b^
	7.00	53.6 ± 0.5 ^a^	10.5 ± 0.4 ^c^	11.7 ± 0.1 ^d^	15.7 ± 0.2 ^c^	48.20 ± 1.38 ^d^	32.3 ± 0.6 ^c^
	10.01	47.7 ± 0.6 ^b^	−8.9 ± 0.8 ^e^	8.9 ± 0.4 ^c^	12.6 ± 0.3 ^d^	134.83 ± 3.99 ^b^	47.9 ± 0.7 ^e^
	12.45	48.0 ± 2.8 ^b^	3.4 ± 0.2 ^d^	19.5 ± 0.9 ^e^	19.8 ± 0.9 ^b^	80.08 ± 0.73 ^c^	37.7 ± 0.4 ^d^
FAPN100	1.68	43.3 ± 1.1 ^a^	31.1 ± 0.8 ^a^	−3.2 ± 0.6 ^a^	31.3 ± 0.8 ^a^	354.14 ± 1.06 ^a^	9.1 ± 0.2 ^a^
	4.01	43.0 ± 0.6 ^a^	19.5 ± 0.5 ^b^	1.4 ± 0.3 ^b^	19.5 ± 0.5 ^c^	4.22 ± 0.99 ^d^	17.8 ± 0.6 ^b^
	7.00	43.6 ± 1.2 ^a^	8.6 ± 0.4 ^c^	6.4 ± 0.2 ^c^	10.7 ± 0.3 ^d^	36.43 ± 1.73 ^c^	29.2 ± 0.6 ^c^
	10.01	43.3 ± 1.0 ^a^	−10.5 ± 0.4 ^d^	8.1 ± 0.2 ^d^	13.3 ± 0.3 ^e^	179.34 ± 0.02 ^b^	48.8 ± 0.3 ^e^
	12.45	43.9 ± 0.6 ^a^	−1.8 ± 0.3 ^e^	21.6 ± 0.3 ^e^	21.7 ± 0.3 ^b^	178.51 ± 0.01 ^b^	42.3 ± 0.1 ^d^

Different letters indicate a significant difference (*p* < 0.05) between means using the Tukey test.

**Table 2:** Moisture content (MC), solubility (*S*), and thickness (T) of films with different polymer proportions.

Formulation	MC (g/100 g)	*S* (%)	T (mm)
FPVA	7.5 ± 0.4 ^a^	98.4 ± 0.3 ^a^	0.10 ± 0.02
FAPN25	6.6 ± 0.5 ^a^	96.1 ± 1.1 ^a^	0.12 ± 0.03
FAPN50	6.9 ± 0.3 ^a^	66.7 ± 1.5 ^b^	0.12 ± 0.02
FAPN75	6.9 ± 0.3 ^a^	52.1 ± 3.4 ^c^	0.11 ± 0.03
FAPN100	6.8 ± 0.2 ^a^	35.1 ± 3.5 ^d^	0.08 ± 0.01

Different letters indicate a significant difference (*p* < 0.05) between means using the Tukey test.

**Table 3:** Tensile strength (TS), elongation (ε), Young’s modulus (YM), contact angle at t = 0 s (CA_0_) and contact angle at t = 30 s (CA_30_) of films with different polymer proportions.

Formulation	TS (MPa)	ε (%)	YM (MPa)	CA_0_ (°)	CA_30_ (°)
FPVA	40.6 ± 6.8 ^a^	277.6 ± 42.5 ^a^	52.1 ± 14.6 ^a^	59.8 ± 9.6 ^a^	59.3 ± 8.9 ^a^
FAPN25	15.9 ± 1.8 ^b,c^	86.1 ± 19.1 ^b^	81.5 ± 28.1 ^a^	78.7 ± 5.8 ^a^	77.0 ± 5.2 ^a^
FAPN50	17.2 ± 1.7 ^b^	63.6 ± 13.2 ^c^	274.1 ± 70.4 ^b^	68.1 ± 11.3 ^a^	68.4 ± 9.2 ^a^
FAPN75	16.4 ± 2.4 ^b^	5.5 ± 0.9 ^d^	978.3 ± 251.7 ^c^	64.8 ± 11.9 ^a^	63.7 ± 9.6 ^a^
FAPN100	13.3 ± 1.3 ^c^	2.6 ± 0.9 ^d^	1127.9 ± 113.2 ^d^	59.3 ± 8.4 ^a^	56.2 ± 7.5 ^a^

Different letters indicate a significant difference (*p* < 0.05) between means using the Tukey test.

**Table 4:** Scanning Electron Microscopy (SEM) images of surface and internal structures of the films.

	FPVA	FAPN25	FAPN50	FAPN75	FAPN100
Surface	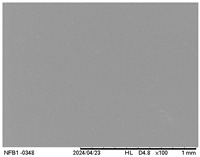	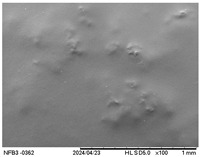	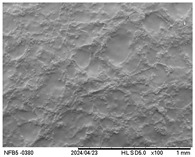	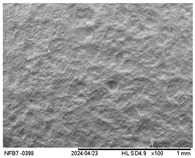	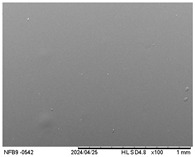
Internal	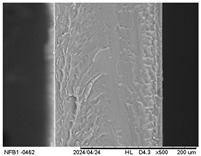	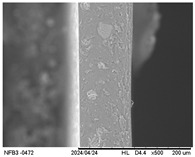	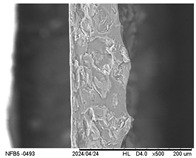	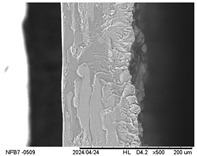	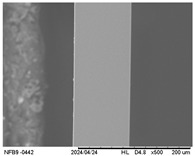

**Table 5:** Color stability of films with different APN ratios at pH 4.01 under various relative humidity conditions over 90 Days (Parameters *L*, a*, b*).

Formulation	L*	a*	b*
FPVA	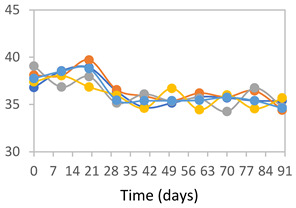	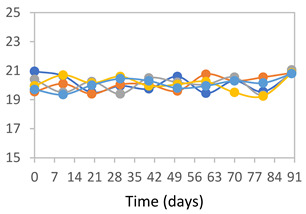	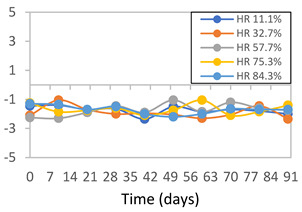
FAPN25	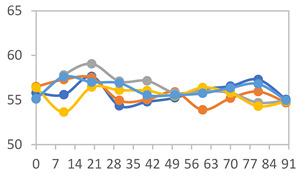	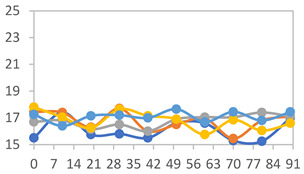	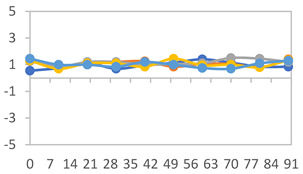
FAPN50	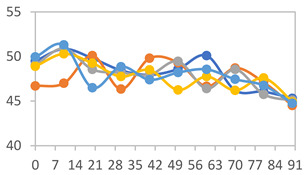	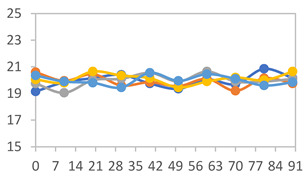	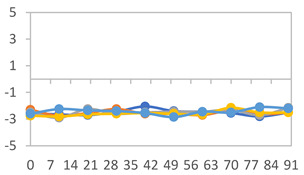
FAPN75	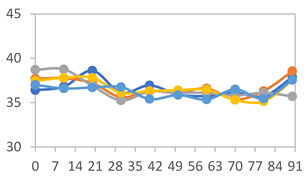	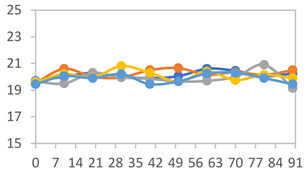	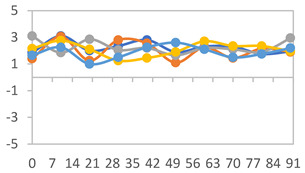
FAPN100	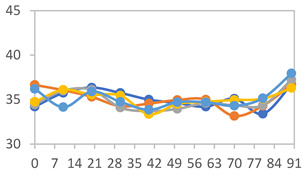	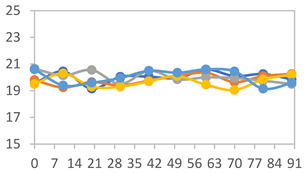	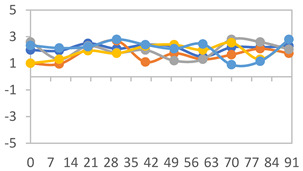

**Table 6:** Color stability of films with different APN ratios at pH 7.00 under various relative humidity conditions over 90 Days (Parameters *L*, a*, b*).

Formulation	L*	a*	b*
FPVA	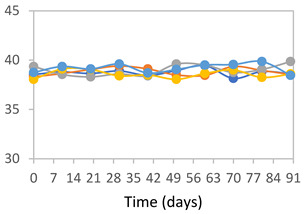	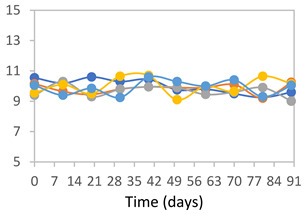	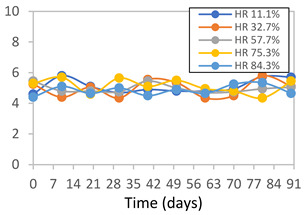
FAPN25	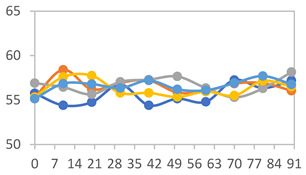	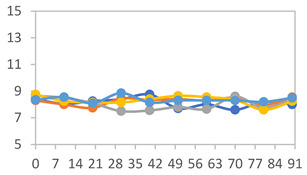	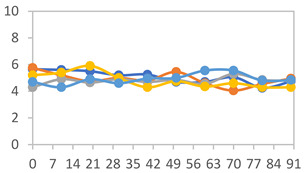
FAPN50	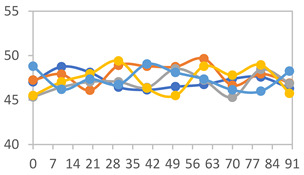	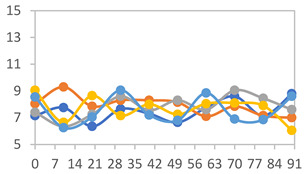	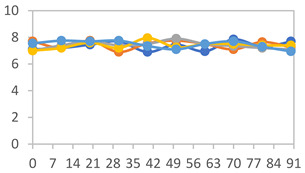
FAPN75	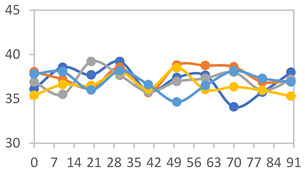	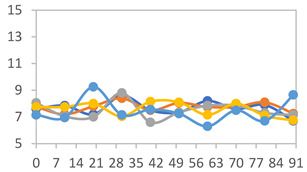	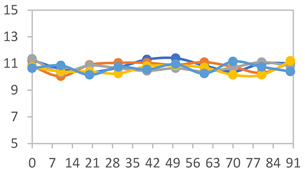
FAPN100	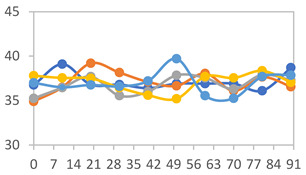	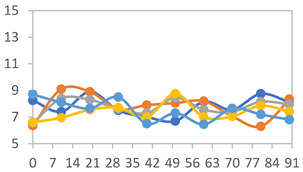	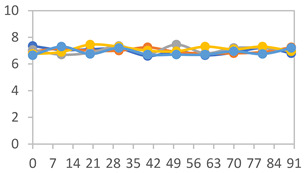

**Table 7:** Color stability of films with different APN ratios at pH 10.01 under various relative humidity conditions over 90 Days (Parameters *L*, a*, b*).

Formulation	L*	a*	b*
FPVA	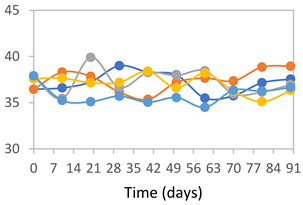	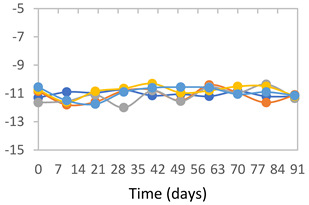	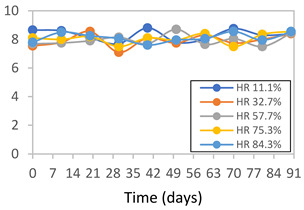
FAPN25	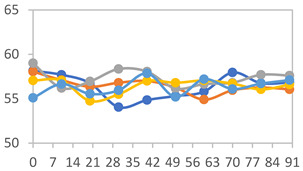	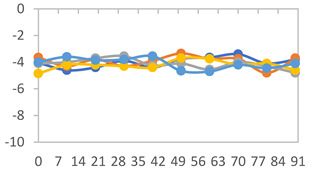	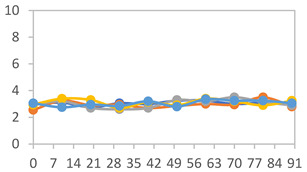
FAPN50	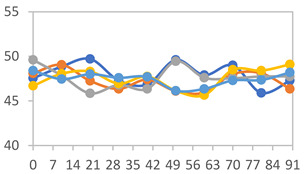	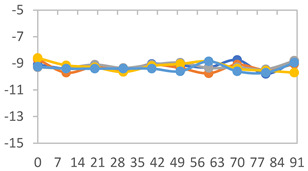	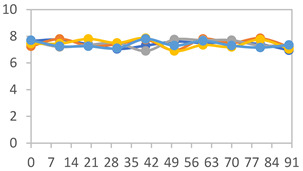
FAPN75	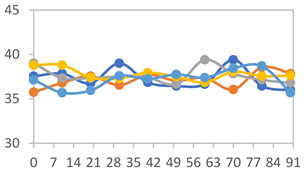	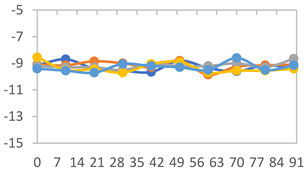	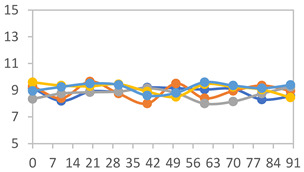
FAPN100	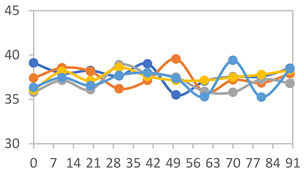	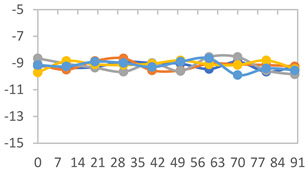	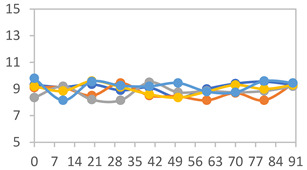

**Table 8:** Changes in color parameters (*L*, a*, b*, c*, h*, and Δ*E*) of films with different proportions for indicating fish freshness over 3 days.

Formulation	Time (h)	*L*	a*	b*	c*	h*	Δ*E* (t = 30 min)
FPVA	0 h	40.7 ± 4.1 ^a^	10.4 ± 1.2 ^a^	−2.9 ± 0.2 ^a^	10.8 ± 1.1 ^a^	354.8 ± 4.4 ^a^	-
	24 h	38.8 ± 2.5 ^a^	8.0 ± 1.6 ^b^	−1.9 ± 0.4 ^a^	8.3 ± 1.5 ^b^	355.8 ± 1.6 ^a^	3.53 ± 2.33 ^a^
	48 h	38.8 ± 1.3 ^a^	−0.6 ± 0.2 ^c^	3.1 ± 0.8 ^b^	3.2 ± 0.8 ^d^	100.3 ± 2.6 ^b^	13.40 ± 1.84 ^b^
	72 h	39.6 ± 1.1 ^a^	−0.3 ± 0.5 ^c^	5.8 ± 2.3 ^c^	5.8 ± 2.3 ^c^	94.0 ± 6.1 ^b^	14.56 ± 2.20 ^b^
FAPN25	0 h	48.0 ± 1.2 ^a^	6.7 ± 0.4 ^a^	−1.9 ± 0.3 ^a^	6.9 ± 0.3 ^a^	354.1 ± 5.6 ^a^	-
	24 h	45.3 ± 0.1 ^a^	4.0 ± 0.1 ^b^	−0.9 ± 0.0 ^b^	4.1 ± 0.1 ^b^	351.5 ± 6.0 ^a^	3.80 ± 0.04 ^a^
	48 h	45.6 ± 0.1 ^a^	−0.4 ± 0.1 ^c^	1.8 ± 0.1 ^c^	1.8 ± 0.1 ^d^	102.6 ± 1.0 ^b^	8.11 ± 0.04 ^b^
	72 h	46.4 ± 0.1 ^a^	−0.4 ± 0.2 ^c^	3.2 ± 0.1 ^d^	3.2 ± 0.1 ^c^	96.3 ± 3.7 ^b^	8.60 ± 0.15 ^b^
FAPN50	0 h	43.0 ± 1.9 ^a^	10.5 ± 1.4 ^a^	−1.8 ± 0.4 ^a^	10.6 ± 1.5 ^a^	353.7 ± 1.3 ^a^	-
	24 h	36.7 ± 4.2 ^b^	10.0 ± 1.3 ^a^	−1.3 ± 0.2 ^a^	10.1 ± 1.2 ^a^	351.3 ± 2.6 ^a^	7.54 ± 2.86 ^a^
	48 h	38.4 ± 2.4 ^b^	2.4 ± 1.7 ^b^	1.2 ± 0.9 ^b^	3.1 ± 0.9 ^c^	33.7 ± 37.2 ^c^	9.98 ± 2.66 ^b^
	72 h	37.4 ± 1.6 ^b^	0.6 ± 0.7 ^b^	5.5 ± 1.3 ^c^	5.6 ± 1.4 ^b^	84.8 ± 5.1 ^b^	13.60 ± 1.65 ^c^
FAPN75	0 h	42.3 ± 1.6 ^a^	7.0 ± 1.5 ^a^	−1.7 ± 0.4 ^a^	7.2 ± 1.5 ^a^	353.8 ± 4.5 ^a^	-
	24 h	36.5 ± 2.1 ^b^	8.2 ± 1.1 ^a^	−1.2 ± 0.3 ^a,b^	8.3 ± 1.1 ^a^	352.8 ± 1.9 ^a^	6.14 ± 1.53 ^a^
	48 h	35.2 ± 0.7 ^b^	1.9 ± 2.0 ^b^	0.6 ± 0.9 ^b^	2.7 ± 0.7 ^b^	157.7 ± 162.2 ^b^	9.56 ± 1.92 ^b^
	72 h	37.0 ± 1.4 ^b^	1.0 ± 0.3 ^b^	6.0 ± 2.1 ^c^	6.1 ± 2.1 ^a^	80.2 ± 4.9 ^b^	11.49 ± 1.46 ^c^

Different letters indicate a significant difference (*p* < 0.05) between means using the Tukey test.

**Table 9:** Changes in color parameters (L, a*, b*, c*, h*, and Δ*E*) of films with different proportions for indicating shrimp freshness over 3 days.

Formulation	Time (h)	*L*	a*	b*	c*	h*	Δ*E* (t = 30 min)
FPVA	0 h	41.1 ± 1.8 ^a^	9.5 ± 2.1 ^a^	−3.0 ± 0.4 ^a^	9.9 ± 2.0 ^a^	353.8 ± 7.5 ^a^	-
	24 h	39.1 ± 2.4 ^a^	8.5 ± 1.4 ^a^	−2.3 ± 0.1 ^a^	8.8 ± 1.3 ^a^	353.7 ± 6.2 ^a^	3.34 ± 2.48 ^a^
	48 h	40.3 ± 1.5 ^a^	1.3 ± 1.1 ^b^	0.5 ± 0.6 ^c^	1.6 ± 0.9 ^b^	34.0 ± 33.5 ^c^	9.14 ± 2.66 ^b^
	72 h	40.6 ± 1.4 ^a^	0.0 ± 0.6 ^b^	3.4 ± 1.9 ^b^	3.5 ± 1.9 ^b^	86.1 ± 15.6 ^b^	11.64 ± 2.89 ^b^
FAPN25	0 h	50.8 ± 2.0 ^a^	7.8 ± 0.8 ^a^	−1.7 ± 1.8 ^a^	8.1 ± 0.8 ^a^	355.7 ± 6.9 ^a^	-
	24 h	49.1 ± 2.2 ^a,b^	6.8 ± 0.6 ^a^	−1.8 ± 0.2 ^a^	7.0 ± 0.6 ^a^	354.7 ± 3.9 ^a^	3.40 ± 2.45 ^a^
	48 h	45.3 ± 2.6 ^b,c^	−0.1 ± 0.2 ^b^	1.8 ± 0.7 ^b^	1.8 ± 0.7 ^c^	96.5 ± 12.5 ^b^	10.42 ± 1.33 ^b^
	72 h	44.5 ± 2.8 ^c^	0.2 ± 0.5 ^b^	3.8 ± 0.4 ^b^	3.8 ± 0.4 ^b^	88.1 ± 7.7 ^b^	11.69 ± 2.13 ^b^
FAPN50	0 h	42.8 ± 1.6 ^a^	9.1 ± 1.4 ^b^	−2.3 ± 0.2 ^a^	9.3 ± 1.3 ^a^	353.8 ± 5.3 ^a^	-
	24 h	39.1 ± 3.2 ^b^	11.8 ± 1.6 ^a^	−1.6 ± 0.2 ^a,b^	11.9 ± 1.6 ^a^	352.4 ± 1.1 ^a^	5.59 ± 2.78 ^a^
	48 h	38.9 ± 2.9 ^b^	4.0 ± 0.6 ^c^	0.6 ± 0.8 ^a,b^	4.0 ± 0.7 ^b^	128.2 ± 176.8 ^b^	7.59 ± 3.06 ^a^
	72 h	39.4 ± 0.6 ^b^	1.1 ± 0.7 ^d^	5.8 ± 3.0 ^c^	5.9 ± 3.0 ^b^	77.1 ± 7.3 ^b^	12.04 ± 2.77 ^b^
FAPN75	0 h	42.1 ± 2.0 ^a^	8.2 ± 1.5 ^b^	−1.5 ± 0.1 ^a^	8.3 ± 1.5 ^b^	353.9 ± 2.1 ^a^	-
	24 h	35.1 ± 1.0 ^b^	10.7 ± 1.7 ^a^	−1.4 ± 0.3 ^a^	10.8 ± 1.7 ^a^	351.6 ± 2.5 ^a^	7.83 ± 2.27 ^a^
	48 h	36.7 ± 1.6 ^b^	5.3 ± 0.7 ^c^	−0.2 ± 0.4 ^b^	5.3 ± 0.7 ^c^	237.9 ± 182.1 ^a^	6.71 ± 2.53 ^a^
	72 h	34.6 ± 1.6 ^b^	0.3 ± 0.2 ^d^	2.5 ± 1.4 ^c^	2.5 ± 1.4 ^d^	82.0 ± 7.0 ^b^	11.94 ± 1.75 ^b^

Different letters indicate a significant difference (*p* < 0.05) between means using the Tukey test.

## Data Availability

Data will be made available on request.
